# Investigation of photocatalytic-proxone process performance in the degradation of toluene and ethyl benzene from polluted air

**DOI:** 10.1038/s41598-023-31183-w

**Published:** 2023-03-10

**Authors:** Jamal Mehralipour, Ahmad Jonidi Jafari, Mitra Gholami, Ali Esrafili, Majid Kermani

**Affiliations:** 1 Department of Environmental Health Engineering, School of Public Health, Iran University of Medical Sciences, Tehran, Iran; 2 Air Pollution Research Center, Iran University of Medical Sciences, Tehran, Iran; 3 Research Center for Environmental Health Technology, Iran University of Medical Sciences, Tehran, Iran

**Keywords:** Environmental sciences, Chemistry

## Abstract

In this study, toluene and ethylbenzene were degraded in the photocatalytic-proxone process using BiOI@NH_2_-MIL125(Ti)/Zeolite nanocomposite. The simultaneous presence of ozone and hydrogen peroxide is known as the proxone process. Nanocomposite Synthesis was carried out using the solvothermal method. Inlet airflow, ozone concentrations, H_2_O_2_ concentrations, relative humidity, and initial pollutants concentrations were studied. The nanocomposite was successfully synthesized based on FT-IR, BET, XRD, FESEM, EDS element mapping, UV–Vis spectra and TEM analysis. A flow rate of 0.1 L min^−1^, 0.3 mg min^−1^ of ozone, 150 ppm of hydrogen peroxide, 45% relative humidity, and 50 ppmv of pollutants were found to be optimal operating conditions. Both pollutants were degraded in excess of 95% under these conditions. For toluene and ethylbenzene, the synergistic of mechanisms effect coefficients were 1.56 and 1.76, respectively. It remained above 95% efficiency 7 times in the hybrid process and had good stability. Photocatalytic-proxone processes were evaluated for stability over 180 min. The remaining ozone levels in the process was insignificant (0.01 mg min^−1^). The CO_2_ and CO production in the photocatalytic-proxone process were 58.4, 5.7 ppm for toluene and 53.7, and 5.5 ppm for ethylbenzene respectively. Oxygen gas promoted and nitrogen gas had an inhibitory effect on the effective removal of pollutants. During the pollutants oxidation, various organic intermediates were identified.

## Introduction

Volatile organic compounds (VOC_s_) are produced in many industrial processes, including pigments, organic chemicals, petrochemicals, and pharmaceuticals. Human's health, especially industrial workers is adversely affected by most VOC_s_. Therefore, VOC_s_ in the ambient or workplace air must be controlled^1,2^. Toluene and ethylbenzene are member of BTEX (benzene, toluene, ethylbenzene, xylene) family which are indicators of VOCs. Individuals and/or industries use toluene extensively, may cause extreme health effects when exposed acutely or chronically. It is well known that toluene can cause respiratory problems in humans, such as chemical pneumonitis, nausea, vomiting, pain, and dermatitis^3^. Cigarettes smoke, gasoline, and natural oil contains ethylbenzene. In addition to affecting the blood, liver, and kidneys, ethylbenzene causes cancer^4^. Currently, many methods have been applied to degradation of BTEX in industry that including adsorption process^5^, catalytic oxidation process^6^, photocatalytic oxidation process^7^, non-thermal plasma process^8^ and biological degradation process^9^. The use of advanced oxidation processes (AOPs) for BTEX removal is a promising approach based on radical generation^10^. One of subsets of AOPs, including the ozonation process (OP) and its derivatives^11^. The ozonation process for BTEX elimination at ambient temperature is favorable as compared to other techniques given energy saving^12^. Auxiliary processes such as photocatalysis^13^, O_3_/H_2_O_2_ (proxone process)^14^, and O_3_/ultrasonic can increase the performance of OP. In the proxone process, the main mechanism of mineralization of toluene and ethylbenzene relies on in-direct oxidation via free oxidation radicals, such as OH^·^, O_2_^·−^ and other radicals^14^. It is also possible to obtain satisfactory mineralization through photocatalysis by adding heterogeneous catalysts into the reaction site and generating electron–hole pairs on the catalyst surface, which is called a photocatalytic process^13^. Recent research has focused on metal–organic frameworks (MOFs) with regular-pore architectures due to their potential applications in gas storage, heterogeneous catalysis, selective adsorption, and sensor technology. MOFs are mainly composed of metal ions or clusters of metal ions, along with organic molecules that act as linkers. Di-, tri-, or tetradendate ligands are typical organic units^15^. Among MOFs, the MIL family is one of the most important. NH_2_-MIL125 is isostructurally identical to MIL-125, but it requires a significantly higher proportion of methanol than DMF for synthesis; it can be prepared by substituting H_2_BDC with 2-amino benzene dicarboxylic acid. It is expected that the amine group in NH_2_-MIL125 will reduce the surface area and the pore size, but the precise position of the amine group in the structure has not been determined^16^. NH_2_-MIL125(Ti) contributes to the photocatalytic degradation of organic pollutants and the evolution of hydrogen because of its suitable band gap. While it exhibits rapid charge recombination, it does not have sufficient structural stability. For improve photocatalytic activity, numerous techniques have been applied, such as substituting mental cations with organic ligands and depositing noble mental cations^17^. BiPO_4_, BiVO_4_, Bi_2_WO_6_, and BiOX (X = Cl, Br, I) are bismuth-containing semiconductors that have been extensively studied for improving photocatalytic and optical properties^18^. Among photocatalysts, BiOI is particularly promising due to its anisotropic layering and suitable band gap. A narrow band gap enables it to respond strongly to visible light^19^. Combining heterojunction structure with MOFs is recommended to overcome fast recombination and stability issues. The large and comfortable pores of Zeolite make it an excellent catalyst or sorbent. A zeolite structure contains Al and Si elements, which provide suitable spaces for trapping pollutants in gas phase^20^. The novelty of this study were, a new BiOI@NH_2_-MIL125(Ti)/Zeolite (BiOI@MOF/Z) nanocomposite synthesization and using as a starting catalyst in photocatalytic-proxone process to toluene and ethylbenzene removal for first time. In this study, the primary objectives were: (i) synthesize a BiOI@MOF/Z nanocomposite that improves the catalytic oxidation process performance for Toluene and EB removal from polluted air, and determine the characterization of the nanocomposite by FESEM, FT-IR, EDS mapping, TEM, XRD, BET, and UV–Vis analysis. (ii), the performance of the Photocatalytic-Proxone Process was examined with respect to parameters (such as flow, ozone concentration, H_2_O_2_ concentration (HP), relative humidity (RH), and initial pollutants concentration), and (iii) to determine the synergy mechanism effect, catalyst stability and reusability, estimating the amount of ozone remaining in the processes, simultaneous toluene and ethylbenzene removal, investigating the effect of oxygen and nitrogen gas as a carrier gas, calculate theoretical mineralization rate of Toluene and EB and release of CO and CO_2_ as well as by-products and probable pathways in optimum condition.

## Methods and materials

### Reagents and materials

Merck, Sigma-Aldrich, and Samchun CO, Ltd. supplied all chemical reagents and materials. The reagents were analytical grade, so no purification was required. *N*, *N* dimethylformamide [DMF, C_3_H_7_NO], 2-aminoterephthalic acid [C_8_H_7_NO_4_], 3-aminopropyl triethoxysilane [C_9_H_23_NO_3_Si (APTES)], tetrabutyl titanate [C_16_H_36_O_4_Ti], ethylene glycol [(CH_2_OH)_2_], bismuth nitrate pentahydrate [Bi(NO_3_)_3_·5H_2_O], potassium iodide [KI], methanol [CH_3_OH], hydrogen peroxide (H_2_O_2_), zeolite (Y model), toluene 99.5% [C_7_H_8_], ethylbenzene [C_8_H_10_], carbon disulfide (CS_2_) were chemicals that used.

### Nanocomposite fabrication

#### MOF model NH_2_-MIL125(Ti)

In the preparation of NH_2_-MIL125(Ti), some changes were made to the amount of material according to the solvothermal approach that had been illustrated in previous literatures^21^. DMF (44 mL) and methanol (6 mL) were mixed with tetrabutyl titanate (1.31 mL) and 2-aminoterephthalic acid (2/1 g). For 20 min at 25 ± 2 °C, the mixture was placed inside an ultrasonic bathroom. A Teflon-lined autoclave was used to heat the mixture solution for 72 h at 175 °C. A final step involves filtering the suspension and washing the solid three times with DMF and methanol, followed by drying at 80 °C.

#### BiOI

As per the literature, BiOI was prepared with a few changes in material amounts^22^. In 15 min, 1.1 mmol KI was ultrasonically dissolved in ml of 50 ml deionized water. A solution of 10 mL of ethylene glycol with 2 mmol Bi(NO_3_)_3_·5H_2_O was then slowly injected into the solution. The suspension was stirred vigorously for 60 min, and then placed in a water bath with a Teflon membrane covering it for 90 min at 70 °C. A final step was to filter, wash, and dry the catalyst at 60 °C in an electric oven several times using DW and C_2_H_6_O.

#### BiOI@NH_2_-MIL125(Ti)

In order to prepare BiOI@NH_2_-MIL125(Ti), a few changes were made in the amount of materials according to the literature^22^. In 50 mL of DW with 1.1 mmol KI, NH_2_-MIL-125(Ti) (0.5 g) was dispersed for 15 min by ultrasonication. The solution was then gently charged with 10 mL of ethylene glycol containing 2.5 mmol Bi(NO_3_)_5_H_2_O. After vigorous stirring for 60 min, the suspension was placed in a water bath with Teflon membrane covered for 90 min at 75 °C. An electric oven was used to dry the catalyst after filtering, washing with DW and C_2_H_6_O several times, and drying at 60 °C.

#### BiOI@MOF/Z

Zeolite was coated with BiOI@NH_2_-MIL125(Ti). Using COOH-modified zeolite, the first composite was synthesized to assess the effect of zeolite surface modification on the performance of the composite. In a slight modification to a previously reported method, –COOH functionalization was carried out^23,24^. A pre-adsorption of humidity was removed from the fine zeolite powder by heating it and degassing it for 120 min under pressure and vacuum at 400 °C. A solution containing 2.5 g succinic anhydride and 10 mL APTES was mixed at 25 ± 3 °C for 30 min, then the solution was dissolved in 50 mL DMF. A further 18 h were spent stirring it with 1.25 g of zeolite. As a result, several washes of ethanol were performed on the resulting powder. It was subsequently dried under vacuum for 24 h at 80 °C. BiOI@MOF/Z nanocomposites were synthesized by modifying seed mediated growth.

#### Characterization of BiOI, MOF, BiOI@MOF, and BiOI@MOF-Z

The FT-IR spectrophotometer (Spotlight 220i FT-IR Microscopy Systems; 4000–400 cm^−1^) used to determine prepared samples functional groups. X-ray diffraction (XRD) patterns of components to assess crystal structure were obtain from XRD diffractometer Rigaku.- ZSX Primus 404; source of radiations: Cu Kα [(λ = 1.54056 Å) monochromatic incident beam in the range of 5° to 80° with the step interval of 0.02°, and rate of 0.05°/s)]. According to the Debye–Scherrer equation, the average size of the crystallites (D) in the nanocomposites can also be calculated (Eq. 1)^25^:

1 $$D=\frac{0.9 \lambda }{\beta {\text{cos}}\theta }.$$

A UV–Visible spectrum was recorded by an Agilent Cary 60 spectrophotometer to investigate structural features and optical properties (UV–Vis DRS). A Tauc formula was used to calculate the band gap (Eq. 2)^26^.

2 $$\left( {{}^\backprime \alpha {\text{h}}\upsilon } \right)^{{2}} = {\text{ A}}\left( {{\text{h}}\upsilon - {\text{E}}_{{\text{g}}} } \right).$$

In FE-SEM (UN41219SEM) the surface morphology of the samples was observed under vacuum conditions of ≥ 1.3 × 10^–4^ mbar. Analyzing purity and elemental mapping of samples was conducted using energy dispersive spectroscopy (EDS). Additionally, a transmission electron microscope (TEM) operated at 200 kV was used to observe the morphology of samples. Catalyst surface area, volume, and distribution pore size were determined at 77 K using a Quantachrome Autosorb analyzer. The catalysts were first degassed in situ for 12 h at 200 °C under vacuum. The Brunauer–Emmet–Teller (BET) to determine linear relations between surface areas was used.

### Configuration for experimental testing

**Figure 1:**
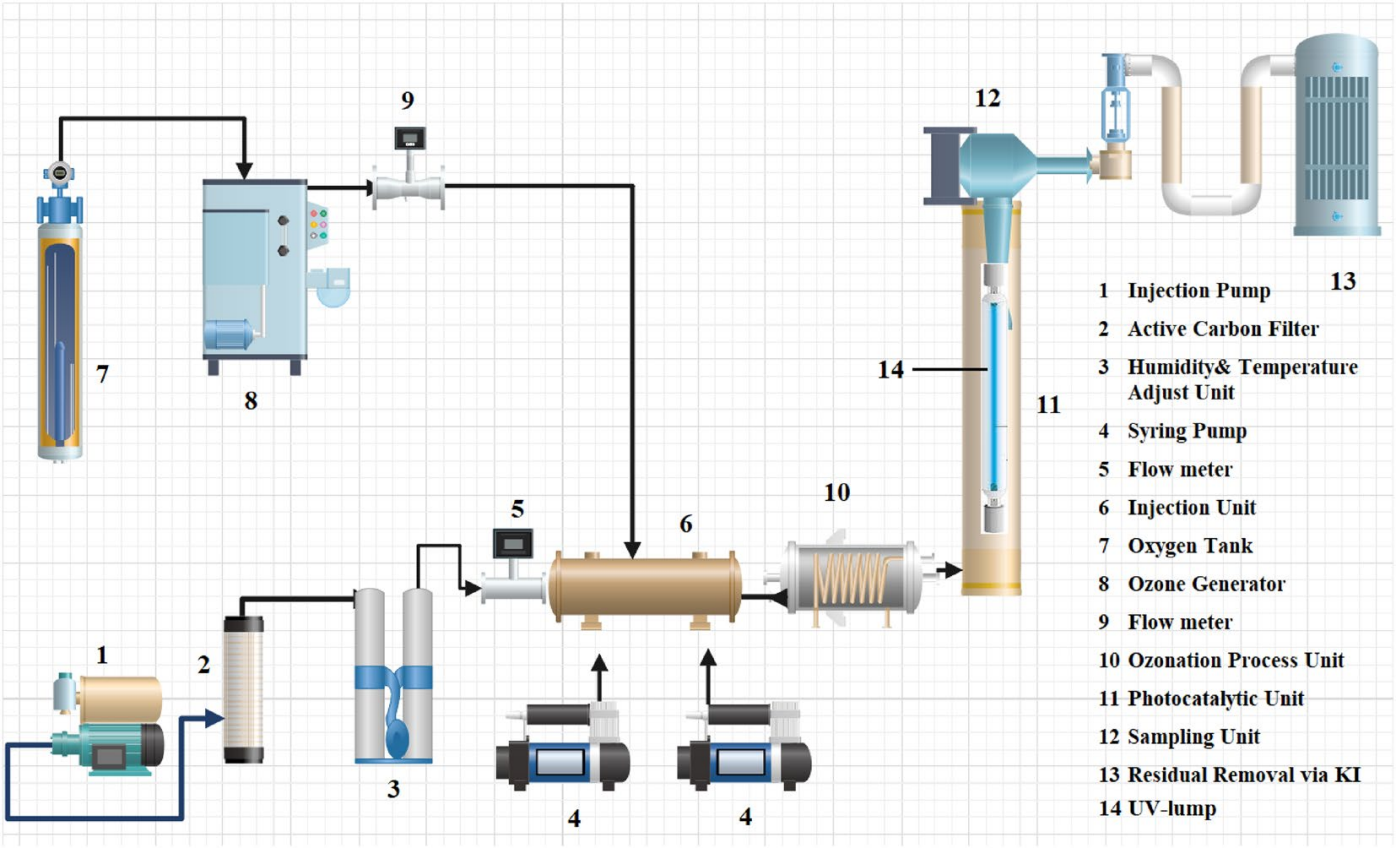
Setup diagram.

There are several components to the experimental setup developed in the laboratory, which include a unit of inlet flow preparation, an ozonation unit, a temperature and RH control unit, an injection unit for pollutants and hydrogen peroxide, and a reaction unit with two main parts: an ozonation process part and a photocatalytic process reactor with vertical cylindrical walls. The photocatalytic site’s active area was 122.46 cm^2^, sampling, and purification unit totalled 221 cm^3^ (Fig. 1). Continuous systems were used for all runs of integrated processes.

A pump (model: A-Q881, 8 W) provided the inlet stream. The used pump had the ability to provide different flow rates and the input flows were adjusted by the flowmeter. In order to remove moisture and probably organic pollutants in inlet airflow, an activated carbon filter and a silica gel column treated the flow current. By using an ozone generator (O180F/DST, Canada), ozone gas is generated. With adjusting the rate of the feed oxygen, the ozone concentration was adjusted. Rotameters controlled the inlet airflow and ozone flow. Ozone concentration was measured by iodometry^27^. An impinger (SKC) equipped with a thermocouple and thermostat provided temperature and relative humidity in the temperature and humidity regulation unit. Two pumps (Avideh Co.) were used to inject toluene, EB and H_2_O_2_. The reactor in photocatalytic part contains a 12-W UV lamp that emits maximum light at 385 nm concentrically next to a quartz tube. As a photocatalyst, BiOI@MOF/Z was supported on the inner surface of the quartz tube with a loading of 1 mg cm^−2^. A potassium iodide (KI) recipient was used to remove residual ozone. Before running the process, sampling was carried out in injection cells to determine the concentration of toluene and ethylbenzene. The degradation process begins with ozone and H_2_O_2_, then streams to the photocatalytic unit via pollutants and ozone inlet, where the degradation process is completed. Additionally, an outlet stream sample was analyzed after oxidation reactions. To determine the effect of operating parameters, flow rate from 0.1 to 3 L-min^−1^, ozone gas concentration from 0.1 to 0.5 mg min^−1^, HP concentration from 50 to 200 ppm, RH from 15 to 55% and initial concentration of pollutants from 50 to 250 ppmv was considered.

### Analytical technique

The toluene and ethylbenzene concentration (ppmv) at both inlet (C_0_) and outlet (C_t_) of reactor was measured based on NIOSH 1501 standard. Samples were collected using charcoal tubes (SKC). Then toluene and ethylbenzene was extracted from the activated carbon using 1 mL carbon disulfide (CS_2_) by ultrasonic bath and measured by gas chromatography (GC-FID) (Agilent 7890A GC System; HP-5 column; 30 m × 0.320 mm × 0.25 µm, American Agilent Company). The steps were repeated three times and the average efficiency is reported. toluene and ethylbenzene removal efficiency was calculated by (Eq. 3)^28^.

3 $${\text{Efficency removal (\% )}} = \left[ {\frac{{{\text{C}}_{{0}} - C_{t} }}{{C_{0}}}} \right] \times 100$$

where, C_0_ and C_t_ are concentrations of toluene and ethylbenzene at the inlet and outlet, respectively.

For the study of intermediate decay, gas chromatography (GC-Agilent 7890A, California, The Unite State) and mass spectrometry (MS-Agilent 5975C) were applied. The DB-5MS column was filled with high-purity (99.99%) helium at a flow rate of 1.0 mL min^−1^ (30 m 0.25 mm 0.5 lm film thickness). As a primer, a 35 °C column temperature was specified for 1 min, increased to 300 °C at 7.0 °C min^−1^, and held for 1 min. A 10 µL sample was injected, and 280 °C was set for the injector and detector. In the optimum of process, calculations of theoretical mineralization rate of toluene and ethylbenzene were shown in (Eqs. 4–7)^29^.

4 $$Mineralization=\left(\frac{{C}_{co2\ outlet}-{C}_{co2\ inlet}}{{C}_{dec.inlet}}\frac{1}{m}\right)\times 100,$$

where, C_CO2_, inlet and C_CO2_, outlet are the CO_2_ inlet and outlet concentrations and m corresponds to the number of carbon atoms in the VOC molecule (toluene = 7, ethylbenzene = 8). CO_2_ emissions were also measured in the outlet using the German Testo 535 carbon dioxide measuring device. A portable CO detector (CO-meter model CO 50, Kimo Instruments, Inc. Canada) was also used to measure CO concentrations^30^. The stability of process in pollutants removal evaluated for 7 runs. The concentration of residual ozone in outlet measurement by the iodometric method^27^. Based on equation (Eq. 5), synergistic effects (SF) between mechanisms were calculated^31^.

5 $$SF = \frac{Hybrid\;Process}{{Sum\;of\;single\;mechanism}}.$$

The efficiency of the process in simultaneous removal of toluene and ethylbenzene under optimal conditions was studied. In this stage, the effect of pure oxygen, nitrogen gases, and atmospheric gas as a carrier of pollutants was investigated.

## Results and discussion

### Photocatalyst characteristics

#### XRD

**Figure 2:**
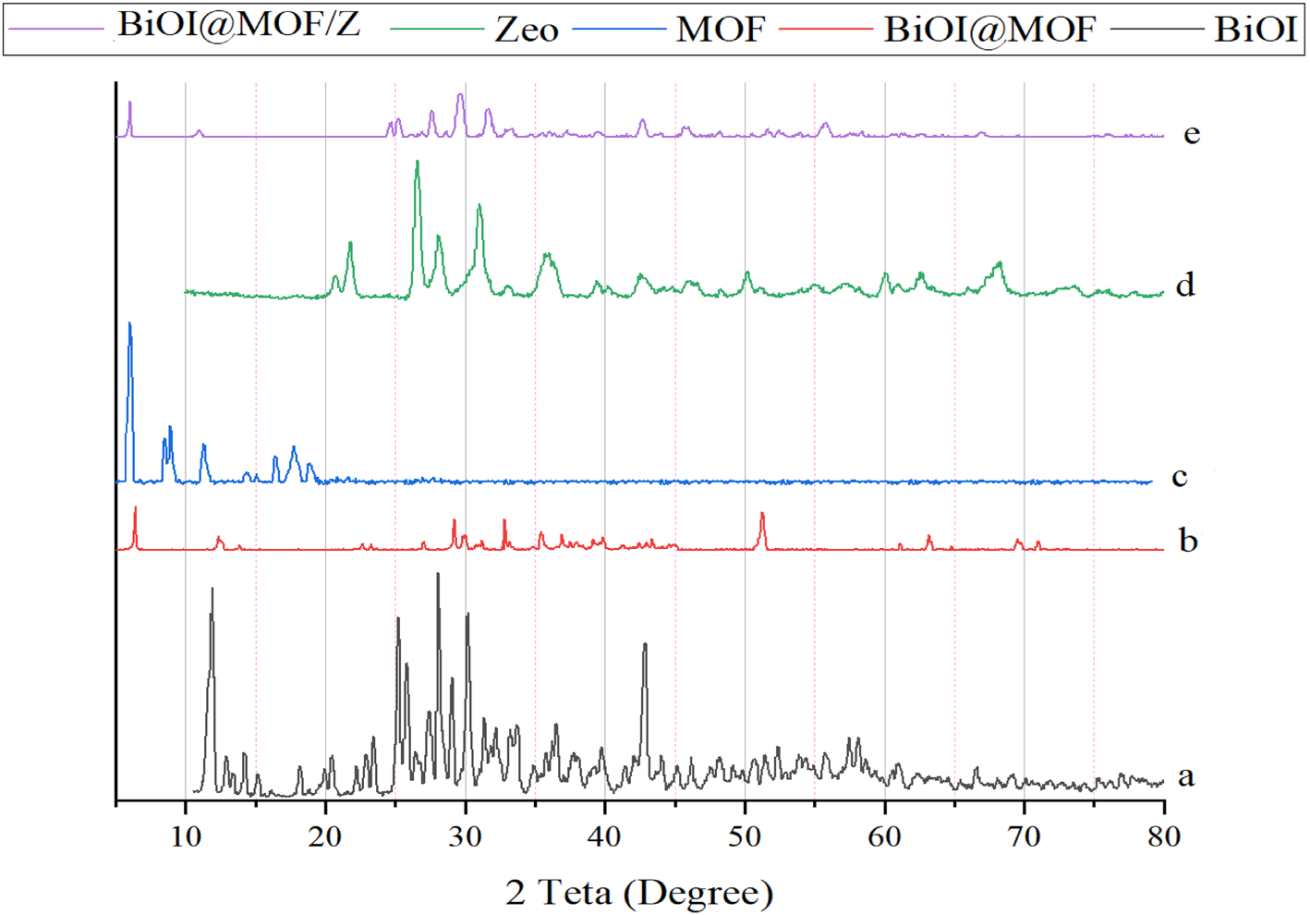
BiOI, MOF, Zeolite, BiOI@MOF, and BiOI@MOF/Z XRD patterns.

The XRD patterns were performed on the prepared samples presented in (Fig. 2).

Five major peaks at (2θ = 12.3°, 25.5°, 28.8°, 31.2°, and 43.2°) located in the XRD pattern of BiOI shown in Fig. 2 a, correspond respectively to the planes (001), (002), (124), (012), and (114). It is clear from the XRD pattern that BiOI belongs to the tetragonal phase, with lattice parameters of a = b 3.994 and c = 9.149, which is noted to be in good agreement with the standard card (JCPDF no. 10-0445). It is evident from the strong and sharp peaks that the as-prepared products have a high amount of crystallinity. According to Liao and coworkers, XRD analysis produced similar results^32^. BiOI@MOF XRD pattern (Fig. 2b), exhibits five characteristic diffraction peaks, but no characteristic diffraction peak was observed for NH_2_-MIL125(Ti), as a result, BiOI@MOF may have a low composition and dispersion. The XRD results of Du et al. study were similar^33^. Based on the XRD pattern (Fig. 2c), MOF crystals were formed, which is consistent with simulated XRD patterns. NH_2_-MIL125(Ti) peaks were located at 6.99°, 9.7°, 12.2°, 17.2°, 18.5°, and 19.7°, which were consistent with MIL-125(Ti) characteristic peaks (013), (113), (215), (324), (211), and (024). NH_2_-MIL125(Ti) showed little effect on its structure when amino groups were present. As indicated by the XRD pattern, the orthorhombic phase is well indexed to the lattice parameters a = 15.03 Å, b = 6.2 Å and c = 19.15 Å. Similar results were reported in Zhang et al. study^34^. Zeolite XRD pattern (Fig. 2d) (JCPDS:43-0168), presents six major peaks at 2θ = 21.7°, 26.5°, 28.1°, 31°, 50.1°, and 60.1° were assigned to the (112), (200), (211), (210), (312), and (150) planes, respectively. In a study by Shoja Razavi and coworkers, similar XRD patterns were reported^35^. Here is an XRD pattern of BiOI@MOF/Z presented in (Fig. 2e). Some of the index peaks present in the base structures have been included in the main structure peaks. Peak intensities were observed. Based on (Eq. 1), BiOI, MOF, BiOI@MOF, Zeolite, and BiOI@MOF/Z crystal sizes are 24, 48, 27.5, 16.5, and 22.6 nm respectively.

#### FT-IR

**Figure 3:**
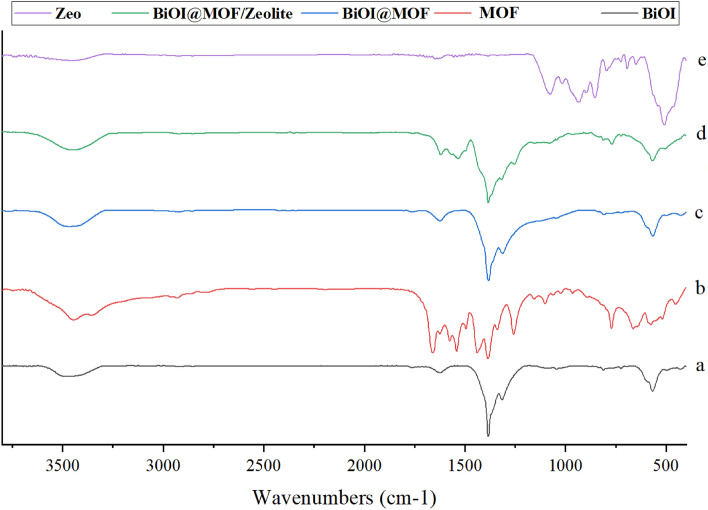
FTIR spectrum of BiOI, MOF, Zeolite, BiOI@MOF and BiOI@MOF/Z.

FTIR spectra of the prepared samples were presented in (Fig. 3).

BiOI exhibits five peaks at 571.23, 1300.904, 1382.38, 1627.08, and 3529.46 cm^−1^ (Fig. 3a). The peak at 571.23 cm^−1^ corresponds to the symmetrical vibration of the Bi–O bond of type A_2_u. At 1300 to 1700 cm^−1^, strong adsorption is observed, along with a large peak at 3175 to 3470 cm^−1^, due to flexural and tensile vibration of the material. According to Han et al. study, a broad peak at 3400 cm^−1^ is caused by O–H vibrations of bonded water molecule, whereas a sharp peak at 530 cm^−1^ is caused by Bi–O stretching^22^. NH_2_-MIL125(Ti) (Fig. 3b) has several peaks in the range of 500–1500 cm^−1^ that are typical of organic compounds. C–N amines have a characteristic tensile strength of 1255 cm^−1^, while the N–H flexural vibration has a characteristic tensile strength of 1622 cm^−1^. There are two obvious peaks at 1535 and 1433 cm^−1^ corresponding to carboxylate bonds. Additionally, the peak around 450 cm^−1^ is attributed to the typical stretching vibration of Ti–O–Ti. Primary amine vibrations also exhibited a broad peak at 4000 cm^−1^, both symmetric and asymmetric. It is not possible to detect any significant vibration bands indicative of the –COOH group in the original molecule between 1600 and 3400 cm^−1^. Similar results were reported in Zhao et al. study. According to this study, major peaks occurred at 773, 1258, 1385, 1539, 1662, 2524, 3059, 3348, 3450 cm^−1^^36^. An FT-IR spectrum for BiOI@MOF (Fig. 3c) is dominated by six major peaks at 565, 807, 1313, 1382, 1624, and 3464 cm^−1^. –CH_3_ bands, C–H bands, and C–H stretch respectively represent alkyl halides, amino acids, N–O nitro compounds, and C–H stretch. According to Han and coworkers, similar results were reported, which further confirmed the formation of BiOI@MOF heterojunctions^22^. BiOI@MOF/Z FTIR spectra (Fig. 3d), exhibits similar peaks to BiOI@MOF. At 550, 1383, and 3450 cm^−1^, three main peaks appeared. A_2_u type Bi–O bond and tension between Si–Al–O cause the peak at 550 cm^−1^. As a result of adsorption of free water molecules on the surface of the material, the peak at 1383 cm^−1^ is attributed to the carboxylate group, while the peak at 3450 cm^−1^ is attributed to flexural vibration (–OH). According to (Fig. 3e), the peaks of the FT-IR spectrum are due to tension between Si and Al in the zeolite. Zeolite frames have a symmetrical tension in the range of 700–900 cm^−1^. Zeolite framework asymmetry produces the peak at 1017 cm^−1^, the NH_4_^+^ functional group produces the peak at 1081 cm^−1^, while the –OH group of water molecules produces the peak at 3470 cm^−1^.

### FESEM, EDS element mapping, and TEM

**Figure 4:**
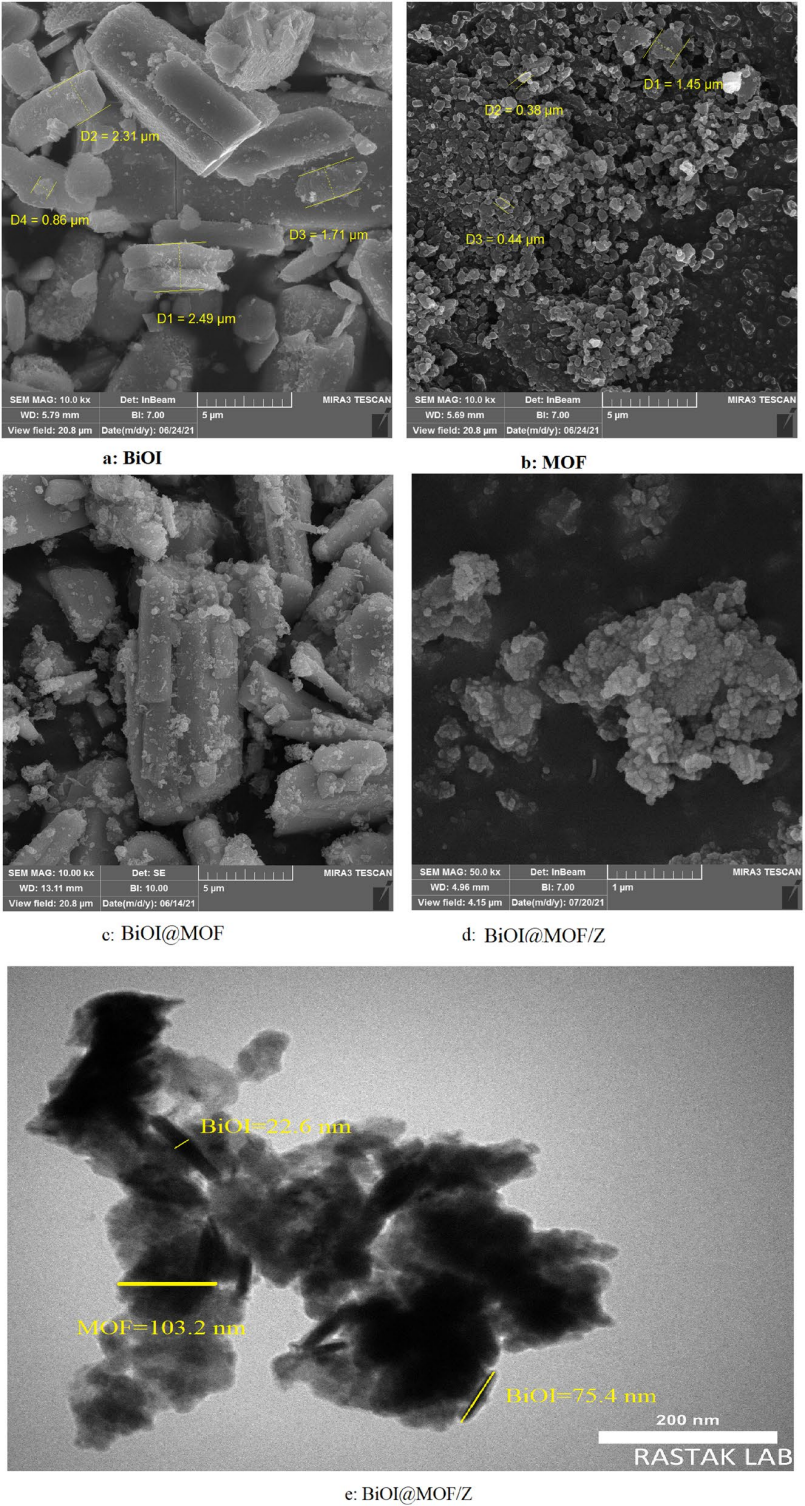
SEM images of (**a**) BiOI, (**b**) MOF, (**c**) BiOI@MOF, (**d**) BiOI-MOF/Z and TEM image of BiOI-MOF/Z.

FESEM images of samples can be found in (Fig. 4).

BiOI, as prepared, displays a micro miller hierarchical morphology (Fig. 4a). BiOI has a rod-shaped structure, as reported by He et al.^37^. A thin disk-like shape was observed for the synthesized NH_2_-MIL125(Ti) (Fig. 4b). Similar FESEM results were reported in Kim et al. study^38^. A rod with fine particles attached to its stem is shown in BiOI@MOF (Fig. 4c). According to Du et al., BiOI@MOF morphology is similar to the morphology reported in their study^39^. The morphology of the BiOI@MOF/Z can be seen in Fig. 4d. The image shows that the catalyst has an asymmetric structure, similar to the natural structure of zeolites. Figure S1, demonstrates the EDS map of the structures synthesized above. Based on these images, it can be seen that the main structural elements are located within the composites. Catalysts contain uniform distributions of these elements. BiOI@MOF/Z TEM image is shown in Fig. 4e. This TEM image shows clearly that MOF/zeolite composite has been successfully distributed on the BiOI surface.

### BET analysis

**Figure 5:**
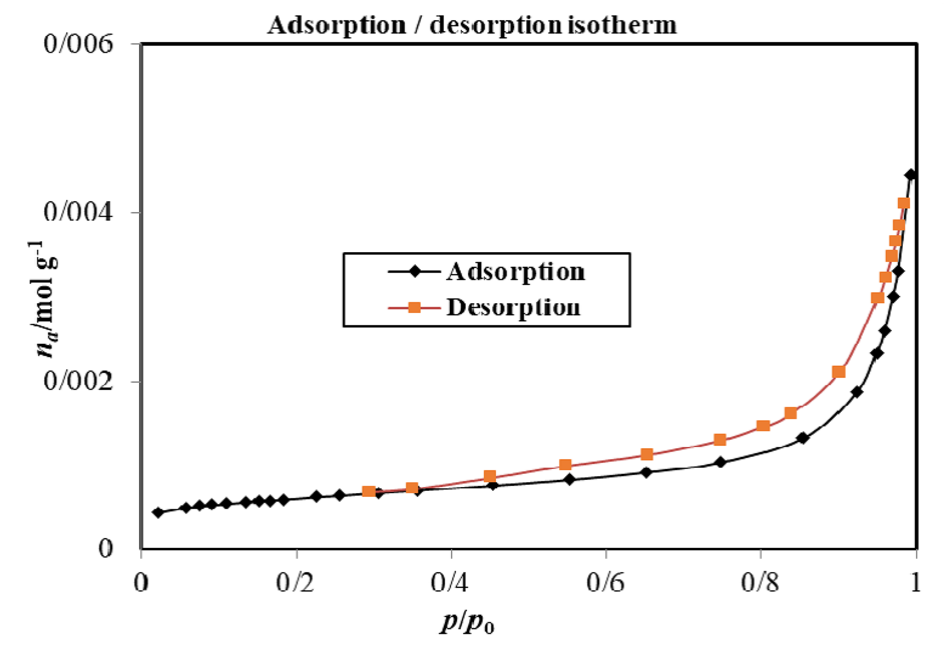
Adsorption/desorption pilot of BiOI@MOF/Z.

The BiOI@MOF/Z, N_2_ adsorption–desorption isotherms are shown in Fig. 5. Type I isotherms with no hysteresis were observed for the sample at 77 K. Barrett–Joyner–Halenda (BJH) method was used to calculate the pore size distribution plot for BiOI@MOF/Z.

Based on Fig. 5, Adsorption rates increase as relative pressure increases, while desorption rates decrease as relative pressure decreases. Hysteresis type IV and model H_3_ can be determined by the curved shape. There are incised cavities and non-hard cavities in type III hysteresis. Tensile strength effect occurs as a result of the steep slope on the H_3_ hysteresis repulsion branch. According to the N_2_ adsorption–desorption isotherm and Barrett–Joyner–Halenda (BJH), the BET surface area and pore volume of the precursor were 947.85 m^2^ g^−1^ and 16.27 cm^3^ g^−1^, respectively.

### UV–Vis spectra, and band gap

**Figure 6:**
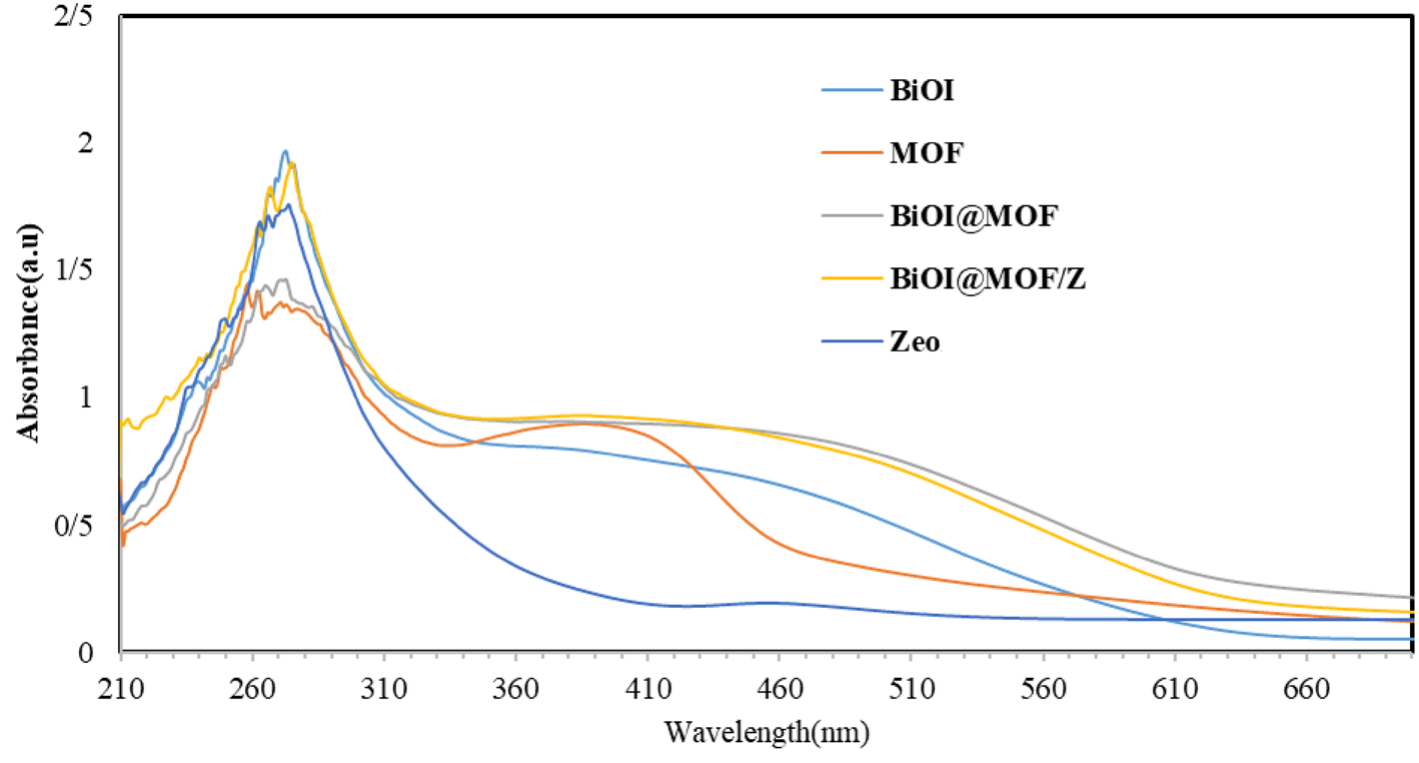
UV–Vis spectra of BiOI, MOF, BiOI@MOF and BiOI@MOF/Z.

Figure 6 shows the UV–Vis spectra of BiOI, MOF, BiOI@MOF, and BiOI@MOF/Z. The band edges are 625, 505, 695, and 680 nm for BiOI, MOF, BiOI@MOF, and BiOI@MOF/Z, respectively.

A gap band was shown in Fig. S2. BiOI@MOF/Z was found to absorb more visible light, which has an effect on electron–hole pair production.

### Performance evaluation of photocatalytic proxone

#### Influence of inlet flow

**Figure 7:**
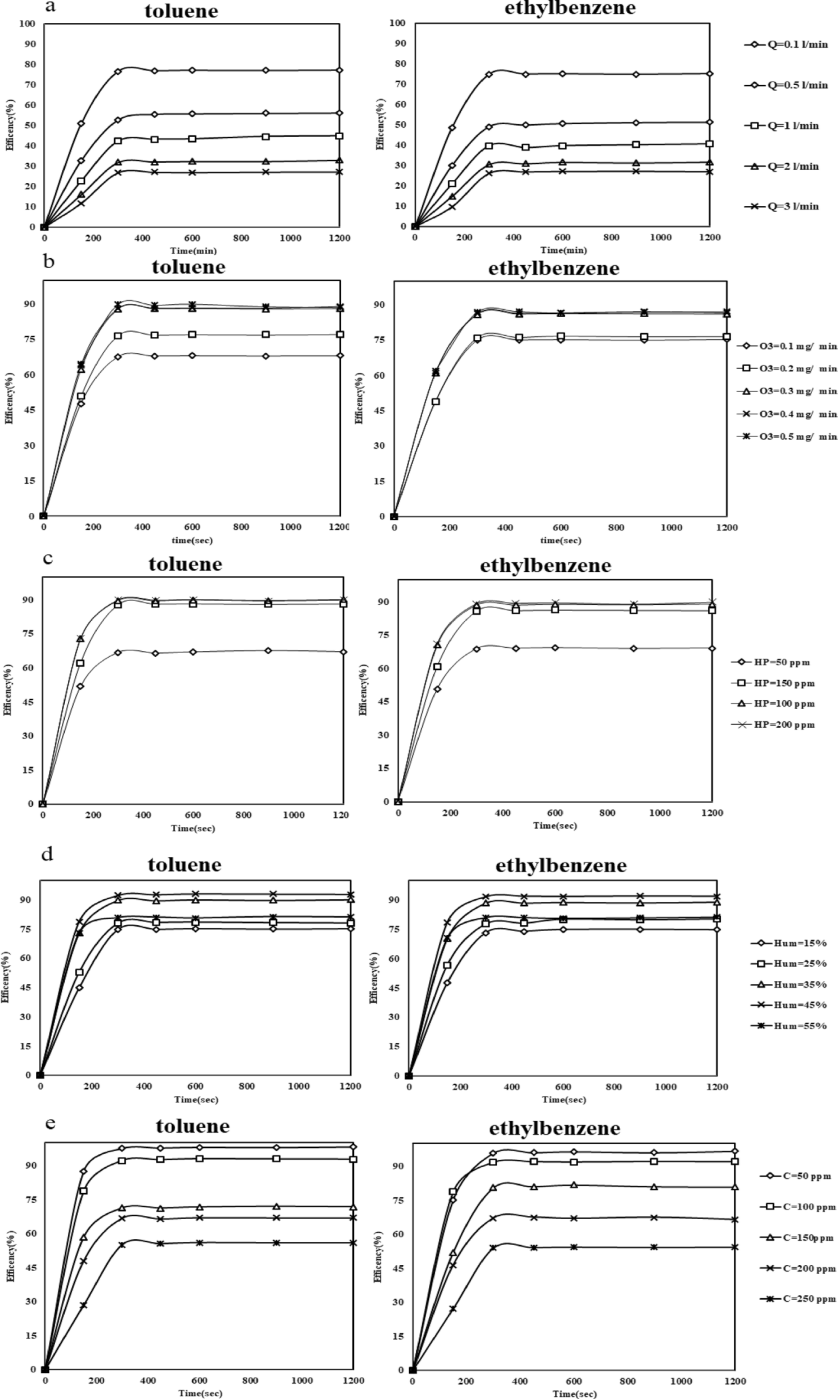
Process parameters affecting toluene and ethylbenzene degradation. (**a**) Air flow (0.1–3 L min^−1^), (**b**) O_3_ concentration (0.1–0.5 mg min^−1^), (**c**) HP concentration (50–200 ppm), (**d**) RH (15–55%), (**e**) pollutants concentration (50–250 ppmv).

The process performance was tested by examining 0.1, 0.5, 1, 2 and 3 L-min^−1^ of inlet air flow. For the above flows, according to the volume of the reactor, the residence times are 101.95, 20.3, 10.19, 5.09, and 3.39 s, respectively. The flow effect on toluene and ethylbenzene degradation is illustrated in (Fig. 7a). The degradation of pollutants was obtained at lowest flow (0.1 L-m^−1^). With an increase in flow, there is less time for chemical reactions between pollutants and oxidizing agents, such as free radicals, ozone, and active oxygen species. A similar result was found in the study by Sangkhun and colleagues. A flow rate of 20 mL-min^−1^ was found to be optimal for BTEX degradation in this study^40^.

#### Influence of ozone gas concentration

Concentrations of 0.1, 0.2, 0.3, 0.4, and 0.5 mg min^−1^ ozone gas were studied to investigated the effect of this parameter on the process performance in toluene and ethylbenzene removal (Fig. 7b). As ozone concentration increases from 0.1 to 0.3 mg min^−1^, removal efficiency increases in the process. Oxidation of pollutants is limited by a some of factors, including (i) the limitations of direct degradation by ozone and (ii) the limitations of the decomposition of ozone into oxidizing radicals and so (iii) the limitations of reaction time to oxidation of pollutants. Equations (6–14) describe the reactions involved in the process^41^.

Direct Oxidation:

6 $${\text{O}}_{{3}} + {\text{organic}}\;{\text{pollutants }} \to {\text{by}}\;{\text{products}} + {\text{ CO}}_{{2}} + {\text{ H}}_{{2}} {\text{O}}.$$

In-direct oxidation

7 $${\text{2O}}_{{3}} + {\text{ H}}_{{2}} {\text{O }} \to {\text{ 2OH}}^{ \cdot } + {\text{ O}}_{{2}} + {\text{ 2HO}}_{{2}} ,$$

8 $${\text{O}}_{{3}} + {\text{ OH}}^{ - } \to {\text{ O}}_{{2}} + {\text{ HO}}_{{2}}^{ - } ,$$

9 $${\text{HO}}_{{2}}^{ - } + {\text{ O}}_{{3}} \to {\text{ O}}_{{3}}^{ \cdot - } + {\text{ O}}_{{2}} ,$$

10 $${\text{HO}}_{{2}}^{ \cdot } \leftrightarrow {\text{ O}}_{{2}}^{ \cdot - } + {\text{ H}}^{ + } ,$$

11 $${\text{O}}_{{2}}^{ \cdot - } + {\text{ O}}_{{3}} \to {\text{O}}_{{3}}^{ - } + {\text{ O}}_{{2}} ,$$

12 $${\text{O}}_{{3}}^{ \cdot - } + {\text{ H}}^{ + } \to {\text{ HO}}_{{3}}^{ \cdot } ,$$

13 $${\text{HO}}_{{3}}^{ \cdot } \to {\text{ OH}}^{ \cdot } + {\text{ O}}_{{2}} ,$$

14 $${\text{O}}_{{3}} + {\text{ OH}}^{ \cdot } \leftrightarrow {\text{ O}}_{{2}} + {\text{ HO}}_{{2}}^{ \cdot } .$$

Catalytic ozonation of toluene over Mn-based catalysts was investigated in Shao et al. study. The results indicated maximum removal of toluene was obtained at 1000 ppm^42^.

### Influence of HP concentration

HP concentrations of 50, 100, 150, and 200 ppm were tested in toluene and ethylbenzene removal efficiency (Fig. 7c). Results showed that the process efficiency increased when HP concentrations increased from 50 to 150 ppm. There have been numerous studies on hydrogen peroxide’s ability to provide hydroxyl radicals. Activating agents such as ozone gas and ultraviolet light cause HP to decompose into hydroxyl radicals. HP concentrations need to be optimized since at higher concentrations than the optimal, by acting as a radical scavenger, it reduces efficiency. In Eqs. (15)–(18), the reactions of the process are shown^43^.

15 $${\text{O}}_{{3}} + {\text{ H}}_{{2}} {\text{O}}_{{2}} \to {\text{ OH}}^{ \cdot } + {\text{ O}}_{{2}} + {\text{ HO}}_{{2}}^{ \cdot } ,$$

16 $${\text{HO}}_{{2}}^{ \cdot } \to {\text{ O}}_{{2}}^{ \cdot - } + {\text{ H}}^{ + } ,$$

17 $${\text{H}}_{{2}} {\text{O}}_{{2}} + {\text{ hu }} \to {\text{ 2OH}}^{ \cdot } ,$$

18 $${\text{O}}_{{2}}^{ \cdot - } + {\text{ O}}_{{3}} \to {\text{ O}}_{{3}}^{ - } + {\text{ O}}_{{2}} .$$

### Influence of RH

RH (15 ± 3, 25 ± 3, 35 ± 3, 45 ± 3 and 55 ± 3%) were investigated to determine the effect on the process efficiency in Toluene and EB degradation (Fig. 7D). When the RH was increased from 15 ± 3 to 45 ± 3%, the process performance in pollutants degradation increased, and then at 55 ± 3% humidity, the efficiency dropped. Based on (Eq. 9), RH is considered as the starter of reaction in AOPs. Hydroxyl radicals are formed by H_2_O during the photocatalytic process. Therefore, humidity is essential in the reaction site. However, increasing the RH leads to a malfunction of the system. Due to the accumulated H_2_O molecules on the surface of the catalyst, it was not possible to fully recover the catalytic activate site in order to decompose O_3_. Also, high RH causes non-uniform input flow. Hong et al. study the catalytic ozonation performance in toluene degradation. In this study, 45% of RH reported as an optimum RH^44^.

### Influence of initial concentration of toluene and ethylbenzene

The effect of initial concentrations of toluene and ethylbenzene on process efficiency was studied at various concentrations (50, 100, 150, 200, and 250 ppmv). When concentration increased 50 to 250 ppmv, the process efficiency decreased (Fig. 7e). Some reasons caused this phenomenon. Since pollutants molecules have a limited reaction time, as their concentration increases, they are less likely to react with ozone or free radicals. (ii) Adsorption of organic matter molecules on the photocatalyst surface is less strong. The degraded products and intermediates of toluene and ethylbenzene also hindered the adsorption to the reactive sites of the photocatalyst when applied at high concentrations to the catalyst. In Jonidi et al. study that investigated the degradation of gaseous toluene from waste air by ozone-assisted photocatalytic, the results indicated that efficiency of process in 50 ppm (lowest concentration) was 87.3% and when toluene concentration increased to 200 ppm, the efficiency of process decreased to 52.6%^45^.

Furthermore, when this process operates at optimum conditions, it is capable of removing over 95% of the initial pollutants (50 ppmv), at a flow rate (0.1 L min^−1^), 0.3 g min^−1^ ozone concentration, 150 ppm of HP concentration, and 45% of RH.

### Effects of mechanisms on photocatalytic-proxone performance

There has been a study of the effectiveness of single, binary, and triple mechanisms. Photolysis (visible light, UV-A and UV-C), ozonation, sorption, and hydrogen peroxide are some of the single mechanisms. A binary mechanism is two mechanisms combination, and a triple mechanism is three single mechanisms combination. According to the results (Fig. S3), the single, binary, and triple mechanisms have less efficiency than the photocatalytic-proxone process. Toluene and ethylbenzene’s synergistic effect was 1.56 and 1.76, respectively (Eq. 8). As a result of the synergistic mechanism, the photocatalytic-proxone process is capable of removing pollutants more efficiently. Conventional ozonation involves the reactions in (Eqs. 6–14). Equation (22) describes the photolysis mechanism. HP causes reactions (15) to (18) at the reaction site. Photocatalysis also generates free radicals, electron pair holes, and superoxide, which oxidize toluene and ethylbenzene indirectly (Eqs. 19–23).

19 $${\text{hu }}\left( {\text{254 nm}} \right) + {\text{toluene and ethylbenzene}} \to {\text{by products}} + {\text{ CO}}_{{2}} + {\text{ H}}_{{2}} {\text{O}},$$

20 $${\text{BiOI}}@{\text{MOF}}/{\text{Z}} + {\text{ UV}} \to \, \left( {{\text{BiOI}}@{\text{MOF}}/{\text{Z h}}_{{{\text{vb}}}}^{ + } } \right) \, + {\text{ e}}_{{{\text{cb}}}}^{ - } ,$$

21 $${\text{e}}_{{{\text{cb}}}}^{ - } + {\text{ O}}_{{2}} \to {\text{ O}}_{{2}}^{ \cdot - } ,$$

22 $$\left( {{\text{BiOI}}@{\text{MOF}}/{\text{Z h}}_{{{\text{vb}}}}^{ + } } \right) \, + {\text{ H}}_{{2}} {\text{O }} \to {\text{ OH}}^{ \cdot } + {\text{ H}}^{ + } ,$$

23 $${\text{O}}_{{2}}^{ \cdot - } + {\text{ OH}}^{ \cdot } + {\text{pollutants}} \to {\text{ CO}}_{{2}} + {\text{ H}}_{{2}} {\text{O}}.$$

### Residual ozone, stability, and reusability of nanocatalysts

**Figure 8:**
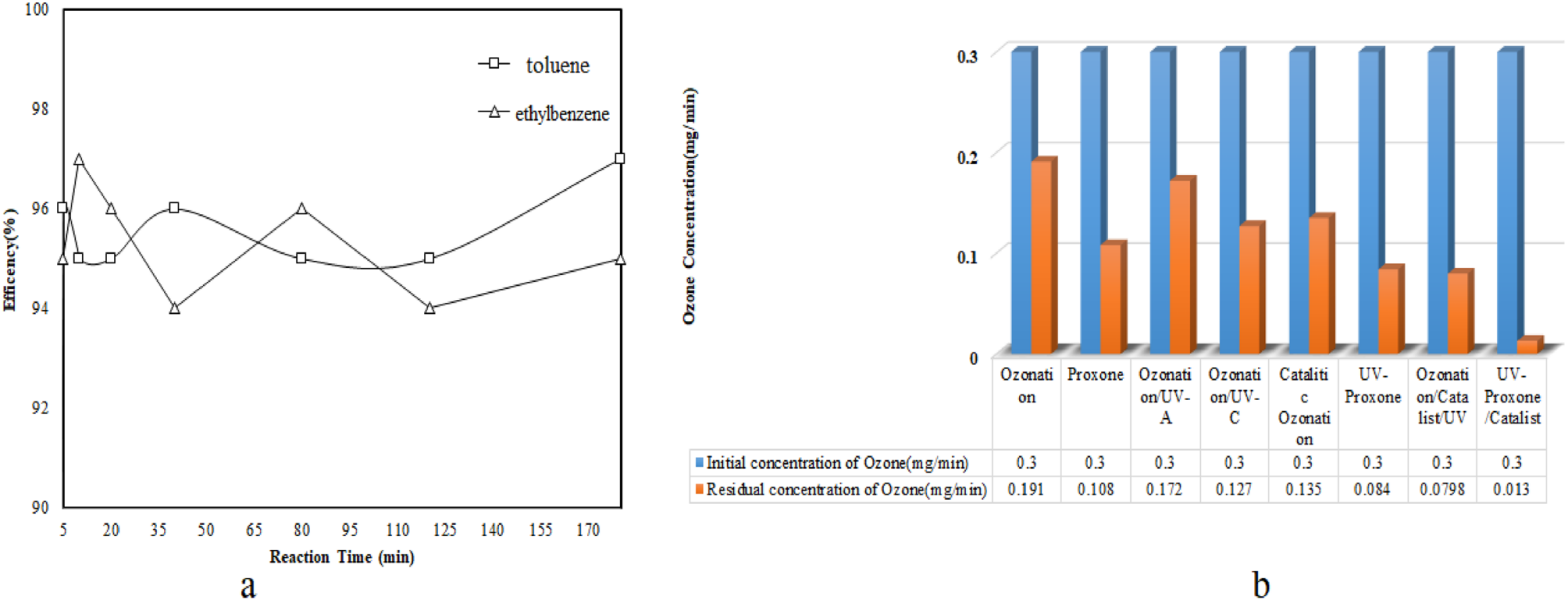
(**a**) Process stability, and reusability of catalyst, (**b**) Residual Ozone in processes (pollutants = 50 ppmv, RH = 45 ± 3, O_3_ = 0.3 mg min^−1^, HP = 150 ppm, and Q = 0.1 L min^−1^).

The stability of process, and reusability of photocatalyst investigated (Fig. 8a). After 7 times of using the catalyst, the process efficiency is still above 95%. BiPO_4_ catalysts have been reported to have long-term stability in photocatalytic systems for degrading benzene by Long et al.^46^. The stability of process in pollutants removal evaluated in 180 min. The results (Fig. 8a) showed the process efficiency after this time of using the process was stable. Wu and coworkers, investigated Toluene degradation by vacuum ultraviolet photolysis and ozone catalysis. Based on results, this process had good stability in toluene removal. In four consecutive stages, process efficiency was reported to be almost constant^47^. As a result of the process, ozone residues play a critical role in determining the direct reaction rate between ozone and pollutant, the rate of ozone decomposition to active radicals, and ultimately the effectiveness of the process. More than 95% of the initial ozone was consumed during the photocatalytic-proxone process (Fig. 8b). Huang et al. report that ozone residues in oxidation processes are negligible, which is consistent with previous studies^48^.

### Simultaneous removal of toluene and EB, and effect of oxygen and nitrogen gases

The process performance in the simultaneous removal of pollutants in optimal conditions was studied. The results showed (Fig. S4a) that in the separate removal of pollutants, the efficiency of the process in removing both pollutant over 95%, but when toluene, and ethylbenzene are present simultaneously, the efficiency of the process was between 60 and 70% of the initial concentration. In a non-thermal plasma catalysis process, Liu et al. remove toluene and styrene simultaneously. The results indicated that with the presence of both pollutants in the reactor, the efficiency of the process decreases and the output of toluene and styrene from the system increased^49^. The effect of pure oxygen, nitrogen gases, and atmospheric gas as a carrier of pollutants investigated in optimum condition. The results indicated that when use of pure oxygen, pure nitrogen, and atmospheric gas, the performance of process changing and the results showed that pure oxygen gas improves and nitrogen gas reduces the efficiency of the process in removing pollutants (Fig. S4b). Chen and coworkers study the effect of N_2_, and O_2_ ratio in 2-chloroethyl ethyl sulphide degradation. The results showed when the plasma performance decreased via increasing the ratio of N_2_ to O_2_^50^.

### Production of CO and CO_2_, intermediate products, and reaction pathway

**Figure 9:**
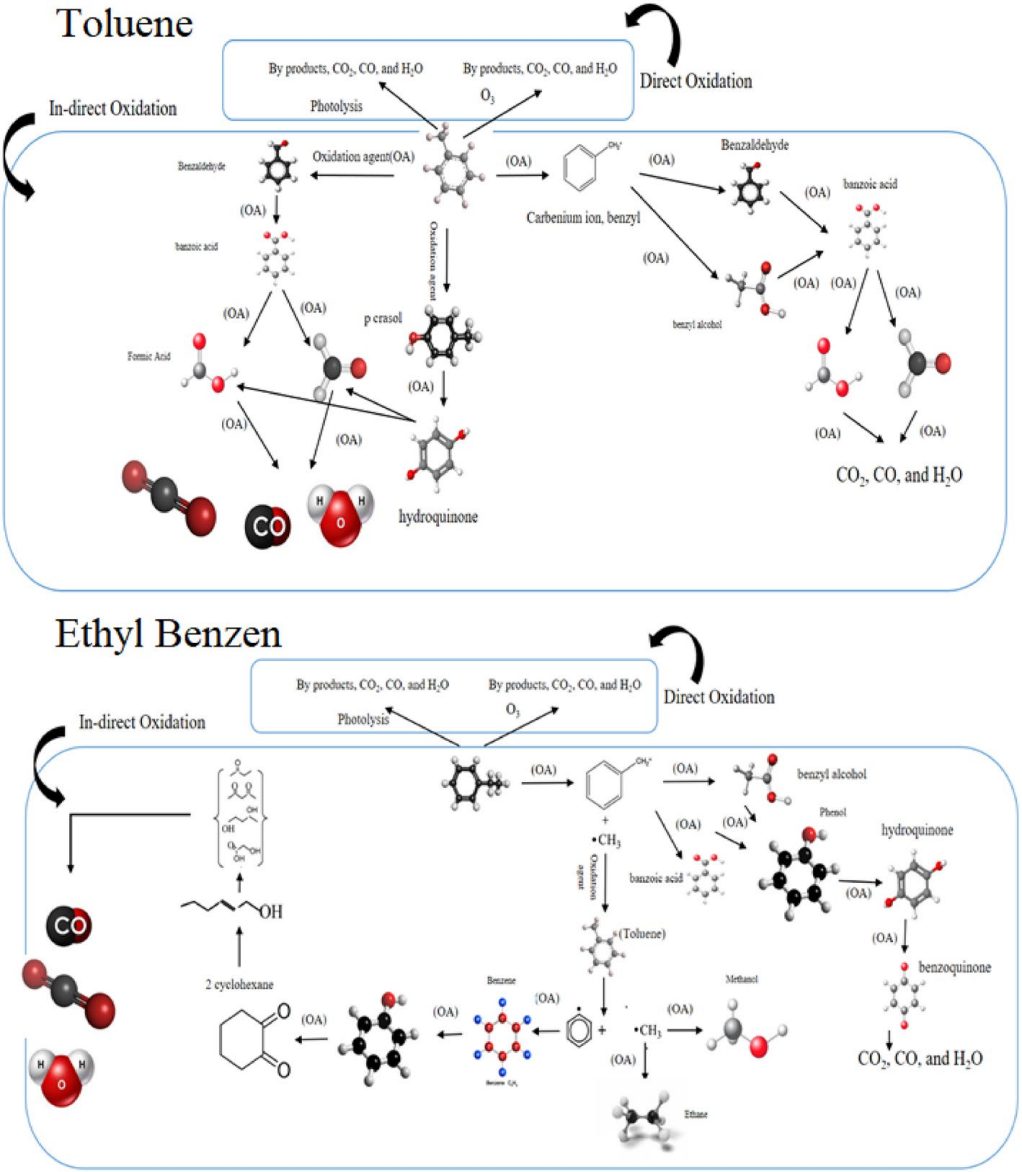
Decomposition probably pathways for toluene and ethylbenzene by photocatalytic-proxone.

CO_2_ and CO production were measured in optimize condition. Production of CO_2_, and CO as a result of successive oxidation of toluene and ethylbenzene. The amount of CO_2_, and CO emissions for toluene was 58.4 and 5.7 ppm, and for ethylbenzene, this value was 53.7 and 5.5 ppm, respectively. Brunet et al. study the emission of CO_2_, and CO gases in catalytic oxidation of toluene. In this study CO reported as a by-product of process^51^. The by-products of toluene and ethylbenzene degradation in the photocatalytic-proxone process were illustrated in (Fig. S5) and the probably reaction pathway were illustrated in Fig. 9. Benzene, formic acid (FA), acetic acid (AA), benzyl alcohol (BA), benzoaldehyde, p-cresol, hydroquinone and benzoic acid are the dominant by-products in the oxidation of toluene by the process. Intermediate compounds resulting from the decomposition of ethylbenzene include toluene, benzene, benzoic acid, benzyl alcohol, benzoaldehyde, cyclohexane, phenol, 2-methylcrotonaldehyde, pentane, hexane and acetone. Direct oxidation by ozone molecules and photolysis and, indirect oxidation by free radicals and electron–hole pairs produced during the process were by two main mechanisms to pollutants degradation.

## Conclusion

A solvo-thermal method was applied to prepare nanocomposite. As shown by XRD, FT-IR, FESEM, TEM, EDS element mapping, BET, and UV–Vis spectra, nanocomposite was well synthesized. In the photocatalytic-proxone process, airflow, O_3_ concentration, HP concentration, RH, and initial toluene and ethylbenzene concentration were studied. It was determined that 0.1 L min^−1^ of airflow, 0.3 mg min^−1^ of ozone, 150 ppm of HP, 45.3% relative humidity, and 50 ppmv of pollutants constitute optimal operating conditions. More than 95% of both pollutants were degraded under these conditions. Toluene and ethylbenzene have synergistic effect coefficients of 1.56 and 1.76, respectively. Up to seven times, the nanocomposite remained above 95% efficiency in the hybrid process. A photocatalytic-proxone process that is stable for 180 min has been investigated. In the system, residual ozone concentrations were reported to be insignificant (0.013 mg min^−1^). In the process, dioxide carbon and monoxide carbon emissions were 58.4 and 5.7 ppm for toluene, and 53.7 and 5.5 ppm for ethylbenzene. Pollutants present at the reaction site reduced the process efficiency. The removal of pollutants was inhibited by nitrogen gas and promoted by oxygen gas. There were several organic intermediates identified during the process of pollutants oxidation. Based on the results, the photocatalytic-proxone process is highly effective in degrading toluene and ethylbenzene as volatile organic compounds.

## Supplementary Information

Supplementary Information.

## Data Availability

The datasets generated and analyzed during the current study available from the corresponding author on reasonable request.

## Abbreviations

AOPs: Advanced oxidation processes
APTES: (3-Aminopropyl) triethoxysilane
BTEX: Benzene, toluene, ethylbenzene, xylene
BiOI@MOF/Z: BiOI@NH_2_-MIL125(Ti)/zeolite
DMF: Dimethylformamide
DW: Distilled water
HP: Hydrogen peroxide
MOF: Metal–organic framework
RH: Relative humidity
VOCs: Volatile organic compounds

## References

[1] Wei L, Yu C, Yang K, Fan Q, Ji H (2021). Recent advances in VOCs and CO removal via photothermal synergistic catalysis. Chin. J. Catal..

[2] Vikrant K, Kim K-H, Peng W, Ge S, Ok YS (2020). Adsorption performance of standard biochar materials against volatile organic compounds in air: A case study using benzene and methyl ethyl ketone. Chem. Eng. J..

[3] Kim K-H (2019). Identifying the best materials for the removal of airborne toluene based on performance metrics—A critical review. J. Clean. Prod..

[4] Derakhshan-Nejad A, Rangkooy HA, Cheraghi M, Yengejeh RJ (2020). Removal of ethyl benzene vapor pollutant from the air using TiO2 nanoparticles immobilized on the ZSM-5 zeolite under UV radiation in lab scale. J. Environ. Health Sci. Eng..

[5] Wang C (2020). Effective removal of aromatic pollutants via adsorption and photocatalysis of porous organic frameworks. RSC Adv..

[6] Li L (2021). Facile synthesis λ-MnO2 spinel for highly effective catalytic oxidation of benzene. Chem. Eng. J..

[7] Liu X, Zhang Y, Matsushima S, Hojo H, Einaga H (2020). Photocatalytic oxidation process for treatment of gas phase benzene using Ti3+ self-doped TiO2 microsphere with sea urchin-like structure. Chem. Eng. J..

[8] Saleem F (2021). Removal of benzene as a tar model compound from a gas mixture using non-thermal plasma dielectric barrier discharge reactor. J. Energy Inst..

[9] Siqueira JPS, Pereira AM, Dutra AMM, Firmino PIM, Dos Santos AB (2018). Process bioengineering applied to BTEX degradation in microaerobic treatment systems. J. Environ. Manag..

[10] Wang J (2021). Applying a novel advanced oxidation process of activated peracetic acid by CoFe2O4 to efficiently degrade sulfamethoxazole. Appl. Catal. B Environ..

[11] Kermani M, Shahsavani A, Ghaderi P, Kasaee P, Mehralipour J (2021). Optimization of UV-electroproxone procedure for treatment of landfill leachate: The study of energy consumption. J. Environ. Health Sci. Eng..

[12] Fang R (2019). Efficient MnOx/SiO2@ AC catalyst for ozone-catalytic oxidation of gaseous benzene at ambient temperature. Appl. Surf. Sci..

[13] Saldanha LAS, Santos NTDG, Tomaz E (2021). Photocatalytic ethylbenzene degradation associated with ozone (TiO2/UV/O3) under different percentages of catalytic coating area: Evaluation of process parameters. Sep. Purif. Technol..

[14] Gorito AM (2021). Ozone-based water treatment (O3, O3/UV, O3/H2O2) for removal of organic micropollutants, bacteria inactivation and regrowth prevention. J. Environ. Chem. Eng..

[15] Yoo DK, Bhadra BN, Jhung SH (2021). Adsorptive removal of hazardous organics from water and fuel with functionalized metal-organic frameworks: Contribution of functional groups. J. Hazard. Mater..

[16] Solís RR, Gómez-Avilés A, Belver C, Rodriguez JJ, Bedia J (2021). Microwave-assisted synthesis of NH2-MIL-125 (Ti) for the solar photocatalytic degradation of aqueous emerging pollutants in batch and continuous tests. J. Environ. Chem. Eng..

[17] Zhang X (2021). Construction of NH2-MIL-125 (Ti)/CdS Z-scheme heterojunction for efficient photocatalytic H2 evolution. J. Hazard. Mater..

[18] Tan HL, Amal R, Ng YH (2017). Alternative strategies in improving the photocatalytic and photoelectrochemical activities of visible light-driven BiVO 4: A review. J. Mater. Chem. A.

[19] Liang Q (2018). Fabrication of BiOI@ UIO-66 (NH2)@ g-C3N4 ternary Z-scheme heterojunction with enhanced visible-light photocatalytic activity. Appl. Surf. Sci..

[20] Alsadun N (2020). Introducing a cantellation strategy for the design of mesoporous zeolite-like metal–organic frameworks: Zr-sod-ZMOFs as a case study. J. Am. Chem. Soc..

[21] Mehralipour J, Jafari AJ, Gholami M, Esrafili A, Kermani M (2022). Synthesis of BiOI@ NH2-MIL125 (Ti)/Zeolite as a novel MOF and advanced hybrid oxidation process application in benzene removal from polluted air stream. J. Environ. Health Sci. Eng..

[22] Han L, Zhang X, Wu D (2019). Construction and characterization of BiOI/NH 2-MIL-125 (Ti) heterostructures with excellent visible-light photocatalytic activity. J. Mater. Sci. Mater. Electron..

[23] Al-Naddaf Q, Thakkar H, Rezaei F (2018). Novel zeolite-5A@ MOF-74 composite adsorbents with core-shell structure for H2 purification. ACS Appl. Mater. Interfaces.

[24] Chen Y (2017). A new MOF-505@ GO composite with high selectivity for CO2/CH4 and CO2/N2 separation. Chem. Eng. J..

[25] Kim SP, Choi MY, Choi HC (2016). Photocatalytic activity of SnO2 nanoparticles in methylene blue degradation. Mater. Res. Bull..

[26] Haque MJ, Bellah MM, Hassan MR, Rahman S (2020). Synthesis of ZnO nanoparticles by two different methods & comparison of their structural, antibacterial, photocatalytic and optical properties. Nano Express.

[27] Siriprom W, Teanchai K, Jitchot N, Chamchoi N (2022). The comparison of ozone production with oxygen concentration and feed gas flow rate at atmospheric pressure. Mater. Today Proc..

[28] Khoramipour S, Mehralipour J, Hosseini M (2021). Optimisation of ultrasonic-electrocoagulation process efficiency in the landfill leachate treatment: A novel advanced oxidation process. Int. J. Environ. Anal. Chem..

[29] da Costa Filho BM (2019). Ozonation and ozone-enhanced photocatalysis for VOC removal from air streams: Process optimization, synergy and mechanism assessment. Sci. Total Environ..

[30] Mao L (2018). Plasma-catalyst hybrid reactor with CeO2/γ-Al2O3 for benzene decomposition with synergetic effect and nano particle by-product reduction. J. Hazard. Mater..

[31] Mehralipour J, Kermani M (2021). Optimization of photo-electro/persulfate/nZVI process on 2–4 dichlorophenoxyacetic acid degradation via central composite design: A novel combination of advanced oxidation process. J. Environ. Health Sci. Eng..

[32] Liao H, Li Z, Luo L, Zhong J, Li J (2021). Water hyacinth powder-assisted preparation of defects-rich and flower-like BiOI/Bi5O7I heterojunctions with excellent visible light photocatalytic activity. Surf. Interfaces.

[33] Du J (2020). The research on the construction and the photocatalytic performance of BiOI/NH2-MIL-125 (Ti) composite. Catalysts.

[34] Zhang Y (2022). Fabrication of NH2-MIL-125 (Ti) nanodots on carbon fiber/MoS2-based weavable photocatalysts for boosting the adsorption and photocatalytic performance. J. Colloid Interface Sci..

[35] Shoja Razavi R, Loghman-Estarki MR (2012). Synthesis and characterizations of copper oxide nanoparticles within zeolite Y. J. Clust. Sci..

[36] Zhao X (2018). NH2-MIL-125(Ti)/TiO2 composites as superior visible-light photocatalysts for selective oxidation of cyclohexane. Mol. Catal..

[37] He R, Cheng K, Wei Z, Zhang S, Xu D (2019). Room-temperature in situ fabrication and enhanced photocatalytic activity of direct Z-scheme BiOI/g-C3N4 photocatalyst. Appl. Surf. Sci..

[38] Kim S-N, Kim J, Kim H-Y, Cho H-Y, Ahn W-S (2013). Adsorption/catalytic properties of MIL-125 and NH2-MIL-125. Catal. Today.

[39] Du J (2021). The research on the construction and the photocatalytic performance of BiOI/NH2-MIL-125 (Ti) composite. Catalysts.

[40] Sangkhun W, Laokiat L, Tanboonchuy V, Khamdahsag P, Grisdanurak N (2012). Photocatalytic degradation of BTEX using W-doped TiO2 immobilized on fiberglass cloth under visible light. Superlattices Microstruct..

[41] Shokri A, Mahanpoor K, Soodbar D (2016). Degradation of ortho-toluidine in petrochemical wastewater by Ozonation, UV/O3, O3/H2O2 and UV/O3/H2O2 processes. Desalin. Water Treat..

[42] Shao J (2020). Low temperature catalytic ozonation of toluene in flue gas over Mn-based catalysts: Effect of support property and SO2/water vapor addition. Appl. Catal. B Environ..

[43] Kermani M, Mehralipour J, Kakavandi B (2019). Photo-assisted electroperoxone of 2, 4-dichlorophenoxy acetic acid herbicide: Kinetic, synergistic and optimization by response surface methodology. J. Water Process Eng..

[44] Hong W (2022). Promoting the catalytic ozonation of toluene by introducing SO42-into the α-MnO2/ZSM-5 catalyst to tune both oxygen vacancies and acid sites. Environ. Sci. Technol..

[45] Jafari AJ, Arfaeinia H, Ramavandi B, Kalantary RR, Esrafily A (2019). Ozone-assisted photocatalytic degradation of gaseous toluene from waste air stream using silica-functionalized graphene oxide/ZnO coated on fiberglass: Performance, intermediates, and mechanistic pathways. Air Qual. Atmos. Health.

[46] Long B, Huang J, Wang X (2012). Photocatalytic degradation of benzene in gas phase by nanostructured BiPO4 catalysts. Prog. Nat. Sci. Mater. Int..

[47] Wu M (2021). Synergetic effect of vacuum ultraviolet photolysis and ozone catalytic oxidation for toluene degradation over MnO2-rGO composite catalyst. Chem. Eng. Sci..

[48] Huang H (2016). Efficient degradation of gaseous benzene by VUV photolysis combined with ozone-assisted catalytic oxidation: Performance and mechanism. Appl. Catal. B.

[49] Liu R, Song H, Li B, Li X, Zhu T (2021). Simultaneous removal of toluene and styrene by non-thermal plasma-catalysis: Effect of VOCs interaction and system configuration. Chemosphere.

[50] Chen D, Peng Y, Gao X, Hou Z (2021). Effects of the N2 to O2 ratio in air on the removal rate and the degree of decomposition of 2-chloroethyl ethyl sulphide by atmospheric plasma. J. Environ. Chem. Eng..

[51] Brunet J (2015). Identification of by-products issued from the catalytic oxidation of toluene by chemical and biological methods. C. R. Chim..

