# Supplementary material for: Investigation of photocatalytic-proxone process performance in the degradation of toluene and ethyl benzene from polluted air
Source: Sci Rep. 2023 Mar 10;13:4000. doi: 10.1038/s41598-023-31183-w (PMC10006189; doi:10.1038/s41598-023-31183-w)
Supplement: Supplementary file 1 — Supplementary Information. [file 41598_2023_31183_MOESM1_ESM.docx]

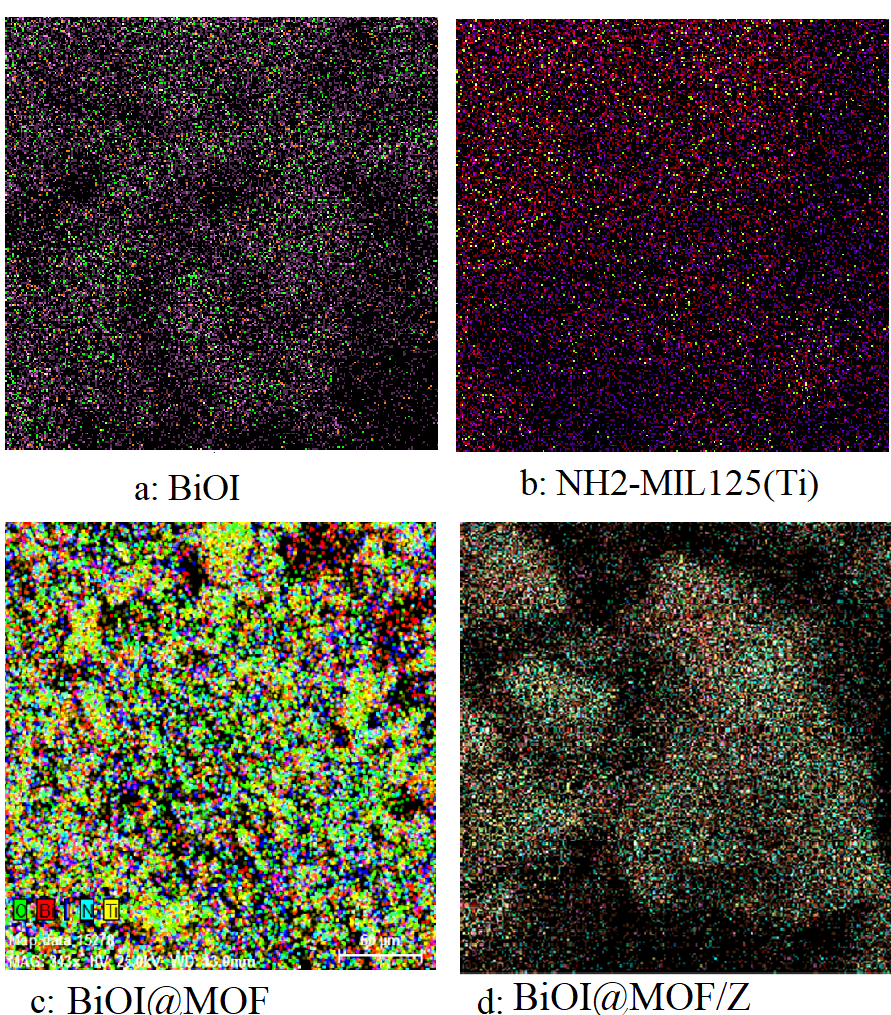


***Fig. S1: EDS mapping of BiOI, MOF, BiOI@MOF and BiOI@MOF/Z***


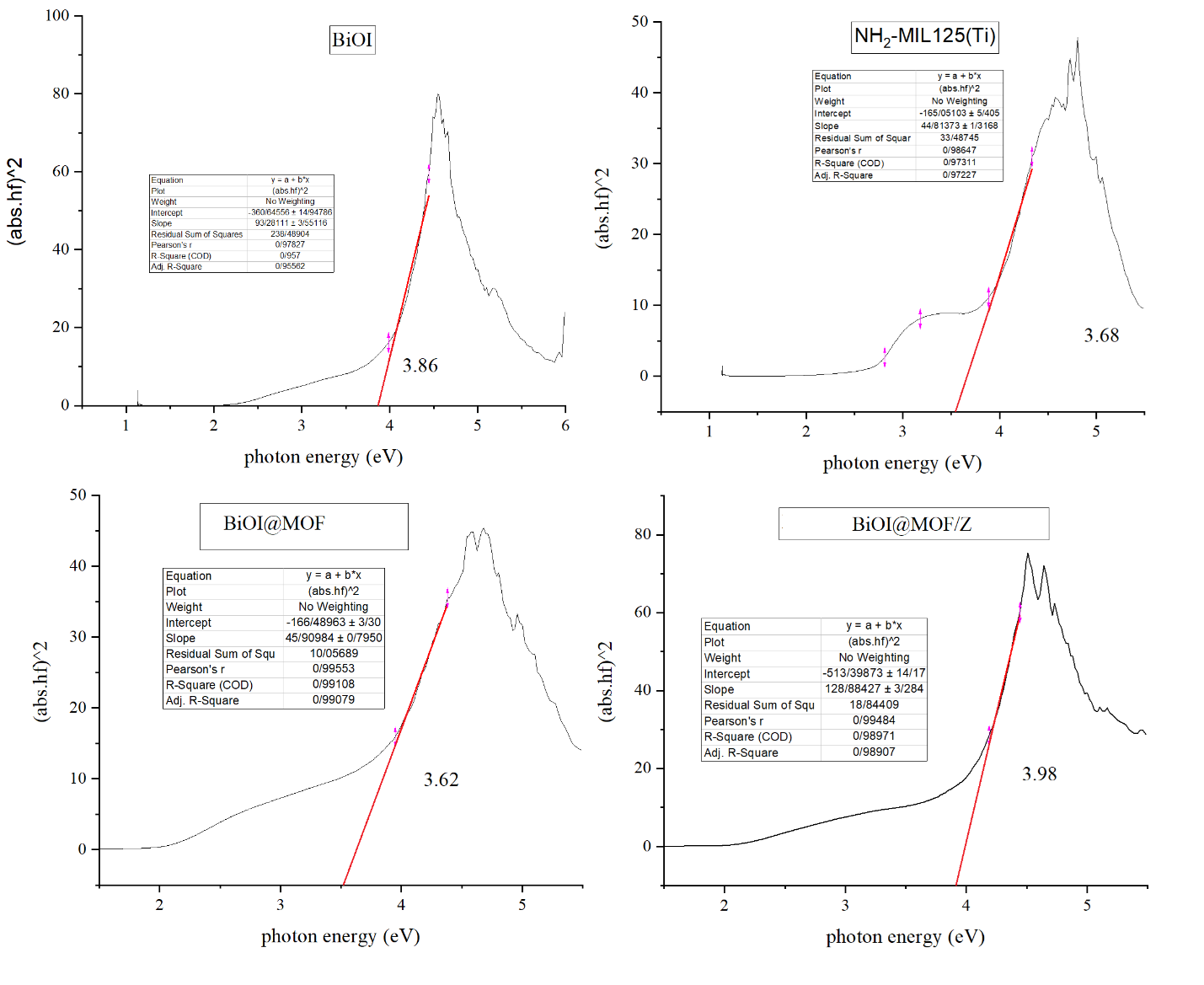


***Fig. S2: Gap band of BiOI, MOF, BiOI@MOF and BiOI@MOF/Z***

***Fig. S3: Single, binary, and triple mechanism effect on toluene and ethylbenzene degradation in optimum condition (pollutants=50 ppmv, O_3_=0.3 mol.l^-11^, HP=150 ppm, RH=45±3, and Q= 0.1 l.min^-1^)***


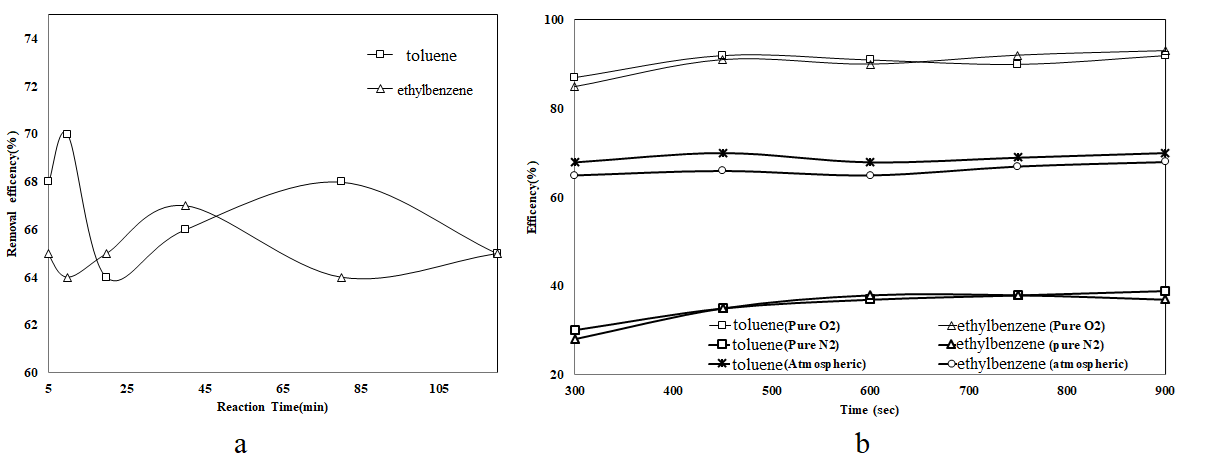


***Fig. S4) a: Simultaneous removal of pollutants, b) effect of the type of gas carrier on the efficiency (pollutants=50 ppmv, O_3_=0.3 mol.l^-1^, HP=150 ppm, RH=45±3, and Q= 0.1 l.min^-1^)***


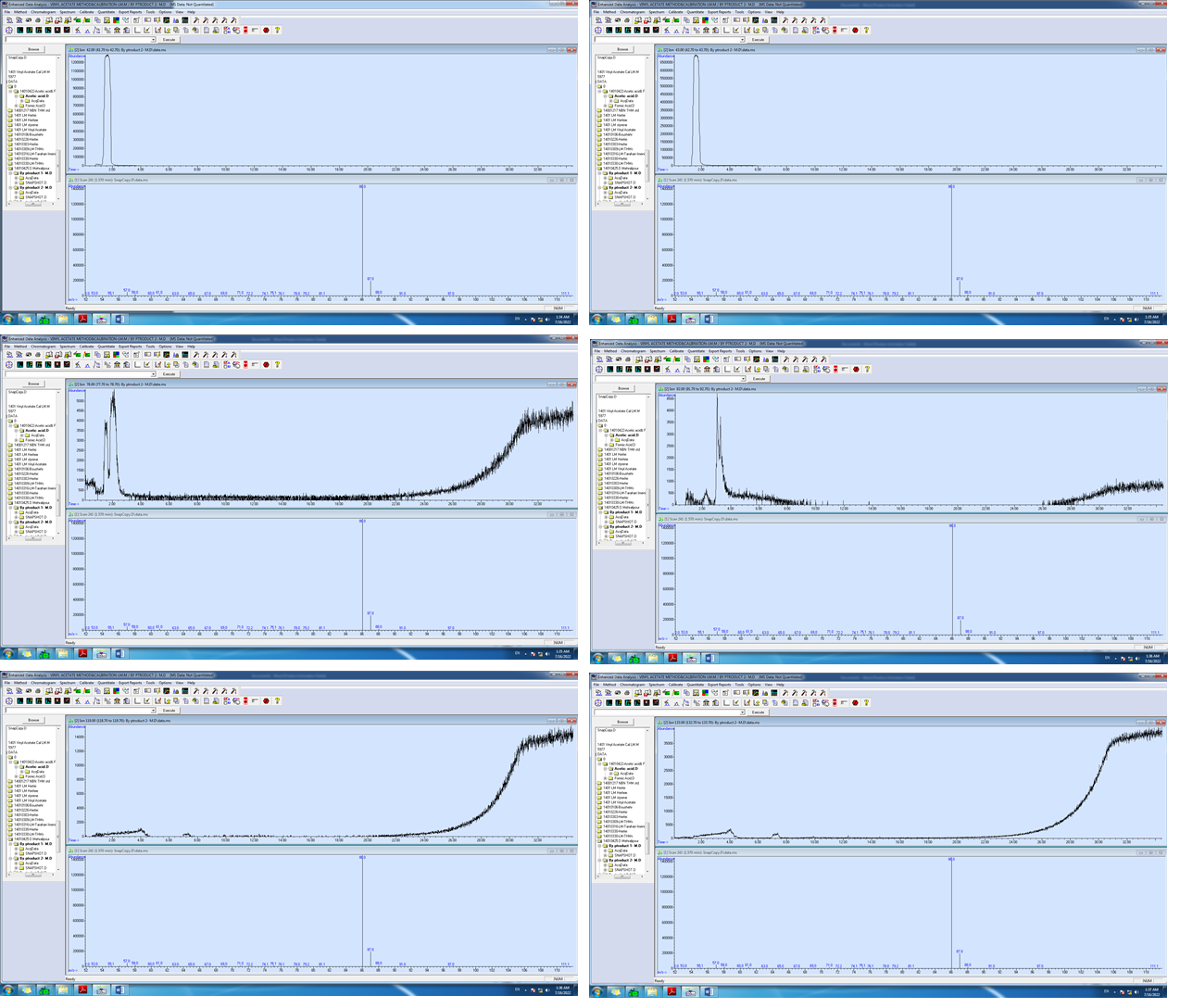


***Fig. S5: GC chromatogram of toluene and ethylbenzene degradation in the photocatalytic-proxone process***
